# Ionic Liquids as Extractants for Nanoplastics

**DOI:** 10.1002/cssc.202001749

**Published:** 2020-09-08

**Authors:** Roman Elfgen, Sascha Gehrke, Oldamur Hollóczki

**Affiliations:** ^1^ Mulliken Center for Theoretical Chemistry University of Bonn Beringstr. 4+6 D-53115 Bonn Germany

**Keywords:** Nanoplastics, Ionic Liquids, Extraction, Molecular Dynamics

## Abstract

Plastic waste in the ocean and on land in the form of nanoplastics is endangering food and drinking water supplies, raising the need for new strategies for the removal of plastic nanoparticles from complex media. In the present contribution we suggest considering ionic liquids as extractants, since they show several advantageous properties that may facilitate the design of efficient separation processes. Through varying the anion and the side chain at the cation, the interactions between the extractant and the polymer can be strengthened and tuned, and thereby the disintegration of the particle into separate polymer chains can be controlled. Oxidized moieties can also be efficiently solvated, given the amphiphilic nature of the considered ionic liquids, allowing also realistic particles to be extracted into these solvents. The phase transfer was found to be thermodynamically and kinetically possible, which is supported by the complicated structure of the ionic liquid‐water interface through the rearrangement of the interfacial ions, and the formation of a micelle around the plastic already at the edge of the aqueous phase.

## Graphical abstract

Hydrophobic ionic liquids are suggested as extracting solvents for the removal of nanoplastics from aqueous systems, to reduce the concentration of these potentially hazardous materials. Based on molecular modeling, comparison with molecular solvents reveal clear advantages for ionic liquids in terms of solvation and disintegration of the plastic particle, and the phase transfer mechanism. The results suggest ways of tuning and improving an extraction process.

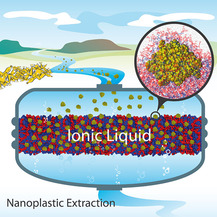

## Introduction

The high amount of plastic waste that is emitted to the environment poses a distinct threat to living organisms, which is presently one of the most pressing ecological issues.^1^, ^2^, ^3^, ^4^, ^5^, ^6^, ^7^, ^8^, ^9^, ^10^, ^11^, ^12^, ^13^, ^14^, ^15^, ^16^ Smaller pieces of plastic, microplastics (MP, <5 mm and >100 nm) and nanoplastics (plastic nanoparticle, PNP, >100 nm) are produced directly to use in diverse costumer products, or originate from physical degradation via mechanical processes or chemical decomposition.^1^, ^10^, ^17^, ^18^, ^19^ In addition, evidence was found that Antarctic krill and potentially other species fragment microplastic beads to nanoplastics in their digestive organs.^20^ Animals take up the plastic particles,^3^, ^4^, ^5^, ^6^, ^7^, ^8^, ^12^, ^13^ and spread them further via the food chain. As a result, plastics have been found in the bodies of various aquatic and terrestrial species.^3^, ^5^, ^6^, ^7^, ^21^, ^22^, ^23^, ^24^, ^25^ We are consuming plastic particles by eating some of these animals, using sea salt,^9^, ^26^ drinking water, and using plastic packaging.^27^, ^28^

There is an increasing number of studies showing potentially adverse effects of nanoplastics, and the findings are rather alarming.^16^, ^29^, ^30^, ^31^, ^32^, ^33^, ^34^, ^35^, ^36^, ^37^ It was recently shown that nanoplastics can enter individual cells.^16^ They can alter biologically relevant properties of lipid bilayers (and thereby cell membranes),^38^, ^39^ while our and other research groups have also shown their effects on proteins as well.^30^, ^31^, ^32^, ^33^ For this reason, it is paramount to establish strategies for the accurate measurement of PNP concentrations, and eventually for the removal of these particles from the environment and from consumption products.^40^ Nguyen et al. recently gave an overview addressing the challenges related to the separation of small plastic particles and their analysis in complex samples.^41^ For the isolation of these particles from such matrices exploiting the properties of plastics that make them different from their matrix is required. Recently, methods using electrosorption^42^ and centrifugation emerged,^43^ which can have applications in purification and analysis in the future. However, up to now there is no general procedure to remove MPs and PNPs, which prompts the need for the development of novel techniques.

In the present contribution we suggest the use of ionic liquids (ILs) as extractants for the removal of nanoplastics from aqueous media. The potential of these liquids lies in their almost infinite variety, allowing functionalization and fine‐tuning of these solvents for any given task. Furthermore, the plethora of diverse interactions within ILs constitute a completely different environment from that of polar or non‐polar molecular solvents. Whereas some ILs are fully miscible with water, some are sufficiently hydrophobic for applications in extracting from an aqueous phase;^44^, ^45^, ^46^ a property, which might be beneficial also for the efficient dissolution of non‐polar compounds that are to be extracted. For these reasons, ILs have been successfully applied for separation and extraction processes of a variety of different materials.^47^, ^48^, ^49^, ^50^, ^51^, ^52^, ^53^, ^54^, ^55^, ^56^ It has been repeatedly demonstrated that, if properly selected, IL‐based solvents and materials can afford higher extraction yields and purification factors as compared to traditional solvents.^50^ Hereby we characterize the solvation of pristine and oxidized polyethylene plastic particles in a set of different ionic liquids, and compare it to molecular solvents with different polarity, in order to identify the advantages of these materials over other possible extractants. We explore the extraction process by simulating the phase transfer, revealing the thermodynamics and mechanism of this process.

## Models and Methods

### Simulated Systems

The present study was performed on a polyethylene nanoparticle (PE‐PNP), composed of 16 chains of C_72_H_144_ as a model system. In order to perform molecular dynamics (MD) simulations, we needed to produce a reasonable starting geometry for the PNP. The molecular structure of these materials is largely unknown, but they are expected to be mostly spherical aggregates of polymer chains in aqueous solutions.^57^ We employed here our previously described^30^, ^31^, ^38^ simulated annealing approach to produce the nanoparticle through a temperature program, involving the slow cooling and eventual nucleation of evaporated polymer chains, and their rearrangement into the desired low‐energy structures.

**Figure 1:**
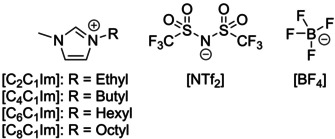
Ionic liquid ions considered in this study.

The polyethylene nanoparticle was surrounded by the solvent molecules with the aid of the program PACKMOL (version 16.228),^58^, ^59^ in order to create the starting geometries for the simulations. Additionally, we performed simulations starting from dissociated polymer chains, which were placed into the simulation box individually and randomly by PACKMOL, to avoid imposing any kind of structure onto the system. The simulation box contained 25000 water molecules, 72 sodium cations and 72 chloride anions in case of saline, which corresponds to the physiological NaCl concentration. The number of solvent molecules in the other systems were: 7500 for ethanol, 5500 for tetrahydrofuran (THF), 5000 for toluene, and 1400 ion pairs for the ionic liquids. The Lewis structures of the ionic liquids are shown in Figure 1. For the simulation of the extraction process we set up an orthorhombic box, which contained two phases, separated along the Z axis. The first phase contained a nanoplastic particle and 24000 water molecules, while the other phase was composed of 1400 ion pairs of either [C_2_C_1_Im][NTf_2_] or [C_8_C_1_Im][NTf_2_].

### Classical MD Simulations

The prepared systems were simulated using the LAMMPS program (version 12^th^ April 2013).^60^ Periodic boundary conditions were applied in order to avoid border effects. For the description of the IL cations and anions intra‐ and intermolecular interactions the force field parameters of Canongia Lopes and Pádua were chosen.^61^, ^62^ For the polyethylene chains as well as for the organic solvents ethanol, toluene and tetrahydrofuran the OPLS‐AA model,^63^ whereas for the simulation of water, the SPC/E water model was used.^64^ In order to obtain Lennard‐Jones cross terms, the Berthelot rule for *ϵ* and the Good−Hope rule for *σ* were applied.^65^, ^66^ All non‐bonded interactions between the atoms were restricted to a cutoff radius of 10 Å, while for larger distances the particle‐particle particle‐mesh long‐range correction was employed. The timestep was set to 1.0 fs. Initial simulations were performed in an NpT ensemble for 7.7 ns at a temperature of 293 K and under a pressure of 1 bar, which was controlled by Nosé−Hoover thermostat and Nosé−Hoover barostat, respectively.^67^, ^68^, ^69^ Over the last 6 ns, the cell volume was averaged, which produced the cell vector that was employed for the subsequent NVT simulations. Table 1 summarizes the physical sizes of the periodic simulation boxes, obtained through NpT simulations. After 1.5 ns of further equilibration in the NVT ensemble at 293 K, for molecular liquids 10 ns while for the more viscous ionic liquids 30 ns production runs were performed, again in the NVT ensemble.

**Table 1:** Cell vectors of the cubic periodic simulation box (in Å) after equilibration in the NpT ensemble for each systems with the globular polyethylene nanoparticle (PE‐PNP), and the dissociated polyethylene chains (PE‐Chains).

Solvent	PE‐PNP	PE‐Chains
Saline	92.1428	92.1478
Ethanol	90.7434	–
Tetrahydrofuran	92.5610	–
Toluene	97.2398	97.2573
[C_2_C_1_Im][NTf_2_]	86.6628	86.6478
[C_4_C_1_Im][NTf_2_]	89.9599	–
[C_6_C_1_Im][NTf_2_]	93.0614	–
[C_8_C_1_Im][NTf_2_]	95.9671	95.9794
[C_2_C_1_Im][BF_4_]	73.4861	73.4464
[C_8_C_1_Im][BF_4_]	86.0987	85.9369

In case of simulating the phase transfer processes, the polyethylene nanoparticle was initially located in the aqueous phase. An additional harmonic force, a bias potential was applied in the system to keep the Z component of the distance vector between the centers of mass of the IL phase and the nanoplastic at 85 Å. Over 6 ns of simulation in the NpT ensemble, the volume of the simulation box was averaged to determine the final cell vectors for the subsequent NVT runs. The system was then equilibrated for another 0.25 ns, followed by 1 ns production run, during which the force applied through the bias potential to keep the nanoparticle in the desired position in the Z direction was averaged. The bias potential was shifted from 85 Å to 0 Å with 1 Å increments, repeating the 0.25 ns equilibration and 1 ns production run at each distance. The integral of the obtained average forces against the distance gave the free energy profile of the extraction.

### Analysis

For analyzing the trajectories we used the TRAVIS software.^70^, ^71^, ^72^ Many of the analyses discussed in the present paper relies on Voronoi analysis. Through Voronoi analysis, all atoms are assigned a cell, and thereby all atoms have volume and surface. The connectivity of these Voronoi cells can be analyzed in a systematic manner, which holds crucial information regarding the spatial distribution (dispersivity) and orientation of the components within the simulation box. The area of contact surfaces between two cells, viz. two atoms, can also be quantified and summarized in a systematic way.

**Figure 2:**
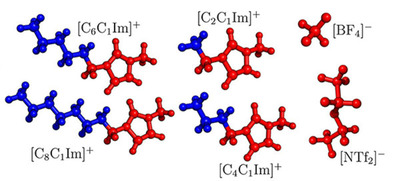
Definitions of the polar (red) and non‐polar (blue) subsets of ionic liquids, as used throughout the article.

Through grouping Voronoi cells that belong to chemically analogous moieties, subsets can be defined, e. g. alkyl groups of IL cations, or ionic components. The connectivity of these subsets can be analyzed by the domain analysis tool of TRAVIS,^72^ which can identify how many continuous domains a given kind of subsets forms. The average of this number over time we will call henceforth domain count *N*. If *N* equals the total number of individual subsets in the simulated system (e. g. 1400 alkyl side chains of the cations), all these subsets are dispersed spatially, and separated by other components of the liquid, whilst any value for *N* smaller than that represents a certain level of aggregation in the system. If the domain count is one, it means that the subsets form a single large, continuous network. The total surface area *A* of a given kind of domain can also be calculated by summing up the contact surfaces of the constituting cells with subsets that do not belong to the domain in question. Next to *N*, *A* can provide another quantitative measure for how dispersed certain subsets or components in the liquid are, and how much the shape of the given domain is altered. The plastic was always considered to be a separate subset, while the definitions of the polar and non‐polar subsets in the ionic liquids are shown in Figure 2.

## Results and Discussion

**Figure 3:**
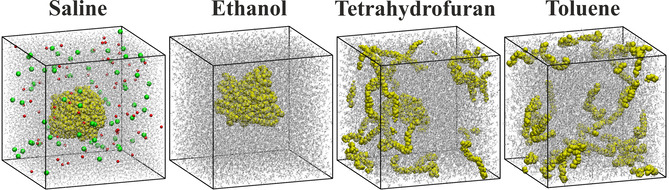
Snapshots of the simulations with molecular solvents (grey) and the polyethylene nanoparticle (yellow) after 10 ns of simulation. The Na^+^ and Cl^−^ ions are depicted in red and green, respectively.

Plastics are often based on non‐polar materials, hence the solvation of PNPs should be highly affected by the polarity of the solvent. To establish a basic understanding on the solvation of PNPs, a polyethylene (PE) particle with a diameter of ca. 5 nm, composed of 16 C_72_H_144_ chains, was simulated in selected molecular solvents that exhibit different polarity: isotonic saline solution, ethanol, tetrahydrofuran, and toluene. When inspecting the geometry of the last snapshot of the trajectories (Figure 3), already at first glance it is apparent that the PNP behaves very differently in these solvents. In water and ethanol, which are the two most polar solvents here, the PE particle retains its globular form, whereas it dissolves in the two non‐polar solvents tetrahydrofuran and toluene. Under closer scrutiny, the particle appears to be very compact in water, thereby minimizing its surface area exposed to the polar solvent. In contrast, the plastic nanoparticle in ethanol shows a notably rougher surface and thus a higher surface area to enable more interactions between the non‐polar PE chains and the non‐polar ethyl groups of the ethanol molecules. However, ethanol still possesses a high ratio of polar moieties, which form an extensive hydrogen bonding network in the liquid, not allowing for a further unfolding and disentanglement of the PE chains. The even less polar solvents tetrahydrofuran and toluene allow for a dissociation of the PE chains in order to maximize the attractive non‐polar‐non‐polar solute‐solvent interactions.

In order to quantify the above described effects, we performed a domain analysis, distinguishing between the solvent and PE, defining the two subsets S and PE, respectively. Table 2 shows the average number of domains *N* emanating from each subset as well as the average surface area of PE covered by the solvent $A^{\mathrm{PE}}$ . The results confirm that within water and ethanol the PE chains stay aggregated and form one single domain. The outer surface area of the PNP is approximately 41 % larger in ethanol than in water, which means that although the chains that constitute the particle stay associated, they loosen their compact structure in order to expose more of the polymers to the solvent.

**Table 2:** Domain analysis^72^ data, describing the solvation of the plastic nanoparticle in molecular solvents. The number of domains composed of the solvent molecules and the polyethylene ($N^{S}$ , and $N^{\mathrm{PE}}$ , respectively) as well as the surface area of the polyethylene $A^{\mathrm{PE}}$ are shown. Of the 10 ns simulation, only the last nanosecond is used for calculating the averages presented here.

System	*N* ^S^	*N* ^PE^	*A* ^PE^ Å^2^
Saline	1.0	1.0	7213.4
Ethanol	1.0	1.0	10167.2
Tetrahydrofuran	1.0	5.6	24938.0
Toluene	1.0	6.8	25409.8

In tetrahydrofuran and toluene the dissociation of the PNP particle into separate chains can be observed through the increased domain counts for the PE ($N^{\mathrm{PE}}$ , Table 2). This effect is notably stronger in the case of toluene, in which $N^{\mathrm{PE}}$ yields a value of 6.8, in contrast to the value 5.6 for tetrahydrofuran. This finding is in agreement with the trends in polarity. Due to this more effective dissociation in toluene, the PE surface area is somewhat larger than in tetrahydrofuran ($A^{\mathrm{PE}}$ , Table 2). Repeating the simulations from the dissociated polyethylene chains gave qualitatively the same result: In saline solution the plastic readily formed a PNP, while in toluene the polymers remained dissociated through the whole simulation.

The trends in the disintegration of the PNP to individual polymer chains as described above unequivocally prove that the non‐polar molecular solvents have more favorable interactions with the PNP. Accordingly, it is very likely that non‐polar solvents can be used for the extraction of pristine PNPs, and these structures dissociate to individual chains within these media. However, such disintegration of the particle might not be advantageous. If the biological decomposition of the plastic is the aim, the fragmentation of the PNPs might be desired, providing better access for digestive enzymes of bacteria to the polymer chains.^73^, ^74^, ^75^ On the other hand, fragmentation creates smaller particles, which are generally believed^16^ – albeit argued^76^ – to be more harmful, if they re‐enter the purified solution in the presence of e. g. surfactants. Furthermore, the aim of the extraction may also be to create a solution with a higher concentration of PNPs from complex environmental samples, which can be analyzed in terms of size distribution, and occurrence.^43^ Obviously, in case of such an application, the disintegration of the particles to individual chains or their aggregation into larger species is disadvantageous. Furthermore, dissolution of the PNP may release compounds that are adsorbed on or absorbed in it,^77^ including drugs, pesticides, plastic additives, which can also pose certain health and environmental risks. For these reasons, it seems vital that the level of PNP disintegration, i. e. the dissociation of the chains that constitute these particles is carefully controlled, adjusted to the problem at hand.

As shown above, the polarity of the solvent has a significant influence on the solvation of the PNP, and probably this feature must be fine‐tuned to fulfill the criteria above. In this regard, ionic liquids (ILs) are promising extractants, since the polarity and hydrophobicity of these compounds can be adjusted easily through varying the anionic and cationic moieties, and the non‐polar side chains. Furthermore, it has been discussed in literature for more than a decade that some ILs possess a unique microscopic structure, called microheterogeneity.^72^, ^78^, ^79^, ^80^, ^81^, ^82^, ^83^, ^84^, ^85^ This phenomenon arises from the mismatch of polar and non‐polar groups of the liquid, which results in the spatial separation of polar and non‐polar domains, sometimes referred to as microphases, within the macroscopically homogeneous liquid. The presence of this peculiar structure has been the source of many intriguing properties of ILs, including their excellent structure directing effect in material synthesis.^84^ For the extraction, this property might be also useful for two reasons. Firstly, the polar and non‐polar domains have a well‐defined structure in any given ionic liquid, with characteristic shapes and sizes.^72^, ^81^, ^83^, ^85^ These pre‐defined dimensions may impose a certain selectivity to the extraction, dissolving only those particles that are small enough to fit into this network of domains. Secondly, PNPs are often not pristine, but they contain – next to the actual polymer – additional compounds. These can be amphiphilic molecules adsorbed onto the PNPs surface^30^, ^86^ (e. g. amino acids with non‐polar side chains^30^), and thereby solubilizing it in a micelle‐like structure, or polar substituents on the polymer chains, produced by the partial oxidation of the PNP surface by air and sunlight^87^, ^88^ or oxidative wastewater treatment.^89^ For weathered macroscopic polyethylene species, both processes have been observed through spectroscopic measurements.^87^ Whilst the former structures can be removed from the PNP during the phase transfer, solvated thereafter separately from the PNP in either of the two phases, the latter ones are covalently attached to the particle, and therefore they must enter the IL phase together with the rest of the PNP. The presence of separate polar and non‐polar microphases within the solution should offer an excellent medium for the simultaneous solvation of both kinds of moieties with different polarity.

**Figure 4:**
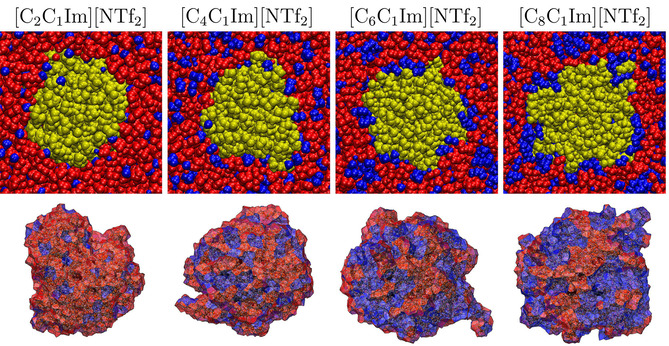
Segments of the simulation boxes containing the polyethylene nanoparticle (yellow) in the different [C_n_C_1_Im][NTf_2_] ionic liquids (polar domain: red; non‐polar domain: blue; see Figure 2), after 30 ns of simulation. Below each segments the voronoi cell of the polyethylene particle is shown, with color indicating the polar (red) or non‐polar (blue) nature of the neighboring moieties at each face of the cell.

**Figure 5:**
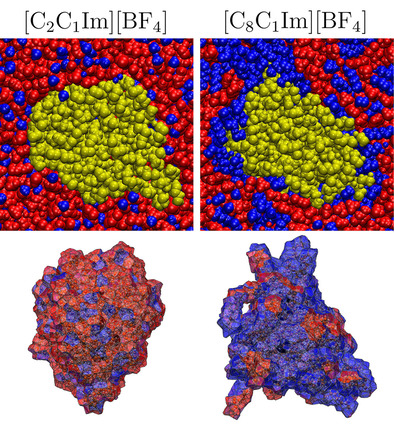
Segments of the simulation boxes containing the polyethylene nanoparticle (yellow) in the different [C_n_C_1_Im][BF_4_] ionic liquids (polar domain: red; non‐polar domain: blue; see Figure 2), after 30 ns of simulation. Below each segments the voronoi cell of the polyethylene particle is shown, with color indicating the polar (red) or non‐polar (blue) nature of the neighboring moieties at each face of the cell.

As model IL extractants for the PE‐PNP, we chose 1‐alkyl‐3‐methylimidazolium bis(trifluoromethylsulfonyl)imide salts, with ethyl, butyl, hexyl, and octyl alkyl side chains ([C_2_C_1_Im], [C_4_C_1_Im], [C_6_C_1_Im], [C_8_C_1_Im], respectively, see Figure 1), which exhibit an increasing degree of microheterogeneity in this order.^72^, ^84^ To vary the anion, we also studied 1‐ethyl‐3‐methylimidazolium tetrafluoroborate and 1‐octyl‐3‐methylimidazolium tetrafluoroborate ILs. The two anions considered here, bis(trifluoromethylsulfonyl)imide and tetrafluoroborate ([NTf_2_] and [BF_4_], respectively), are both hydrophobic, making the corresponding ILs largely immiscible with water.^44^ Visible already at the segments of the snapshots shown in Figures 4 and 5, by the increase in side chain length the polar (red) and non‐polar (blue) domains become more segregated in the liquid, as observed previously.^72^, ^78^, ^79^, ^80^, ^81^, ^82^, ^83^, ^84^, ^85^ This is fully corroborated by the domain analysis (Table 3), which shows that while the polar domain includes all polar subsets into a single network of ions in all systems, the number of non‐polar domains ($N^{N}$ ) decreases from 667.1 in case of [C_2_C_1_Im][NTf_2_] to 2.8 in case of [C_8_C_1_Im][NTf_2_]. It is worth mentioning here that the number of non‐polar domains have been found to be $N^{N}=1.0$ for the neat ionic liquids with a side chain length longer than a butyl group,^72^, ^84^ which shows that the nanoplastic separates some of the solvating ions from the non‐polar domain, and creates its own environment within the liquid. Due to the smaller size of the anion, in the [BF_4_]‐based ionic liquids the non‐polar moieties can form larger, more uninterrupted domains in the liquid than in the corresponding [NTf_2_] derivatives (cf. $r^{N}$ and $N^{N}$ in Table 3).

**Table 3:** Domain analysis data^72^ for the systems containing the ILs and the polyethylene nanoparticle. The average volume percentage of non‐polar domains in the liquid (*r* ^*N*^), the average number of polyethylene (*N* ^PE^), polar (*N* ^P^) and non‐polar domains (*N* ^N^), as well as the average total outer surface area of the polyethylene particle (*A* ^PE^), and the average surface area of the plastic particle covered by the non‐polar domain of the ionic liquid are (*A* ^PE*/*N^) presented, together with literature viscosity (*η*). Only the last ns of the 30 ns simulation was used to calculate the averages.

System	*r* ^N^ %	*N* ^PE^	*N* ^P^	*N* ^N^	*A* ^PE^ Å^2^	*A* ^PE*/*N^ Å^2^	*η* mPa s
[C_2_C_1_Im][NTf_2_]	9.45	1.0	1.0	667.1	8211.0	1331.6	40^*a*^
[C_4_C_1_Im][NTf_2_]	20.10	1.0	1.0	89.1	8766.7	3261.6	63^*a*^
[C_6_C_1_Im][NTf_2_]	29.03	1.0	1.0	15.1	9503.8	5208.7	90^*a*^
[C_8_C_1_Im][NTf_2_]	40.18	1.0	1.0	2.8	10376.5	6707.2	120^*a*^
[C_2_C_1_Im][BF_4_]	14.60	1.0	1.0	303.9	8697.7	2440.4	67^*b*^
[C_8_C_1_Im][BF_4_]	51.81	1.0	1.0	1.7	11651.2	9348.9	439^*b*^

^*a*^ experimental values taken from Ref [90], ^*b*^ experimental values taken from Ref [91]

The solvation of the PNP varies in the different ILs. The PNP is apparently encompassed by the non‐polar domain, and the proportion of the surface covered by the non‐polar moieties of the solvent increases significantly toward the ILs with longer cationic side chains (Figures 4 and 5, and Table 3). Although the polyethylene chains that build the particle remain associated throughout the simulations (see $N^{\mathrm{PE}}$ in Table 3), the particle strongly reacts to the presence of the IL, as shown by the changes in its total surface area $A^{\mathrm{PE}}$ (Table 3). $A^{\mathrm{PE}}$ is larger compared to that in water already in the most polar [C_2_C_1_Im][NTf_2_], and increases steadily toward the less polar ILs with longer side chains up to the octyl derivative. In Figures 4 and 5 it can also be observed that the unfolding of the polymer chains from the particle occurs at those positions, where the non‐polar domains are in contact with the PNP. The highest surface area of the particle is similar to that in ethanol. These observations are consistent with stronger solute‐solvent interactions of the PNP within the IL than within an aqueous solution, suggesting a higher solubility of the PNP in the IL than in water. In accordance with the trends in the size of the anions, and in the corresponding volume ratio of the (for the plastic apparently more attractive) non‐polar moieties in the liquid ($r^{N}$ , Table 3), the 1,3‐dialkylimidazolium tetrafluoroborate derivatives show higher surface areas than those with the [NTf_2_] anion, indicating a more intensive interplay between the particle and the solvent.

Thus, the trends shown in the *N* and *A* data in Table 3 seem to be reasonable, as they represent a good correlation between the degree of rearrangement of the PE chains within the PNP and the polarity trends of the IL solvents. However, there are some limitations of MD simulations, which are worth pointing out in the present context. A statistically meaningful description of the transition between two states – e. g. the globular nanoparticle, and the dissolved, dissociated PE chains – can be achieved only if both states are sampled. In practice, this means that the transition from one state to the other – in this case the dissolution/disintegration process – occurs within the time frame of the simulation. ILs are viscous solvents (see Table 3), which should lead to a relatively slow mass transport within the liquid. In classical MD simulations the diffusion of the ions is known to be underestimated,^92^ making a dissolution process in these model systems even slower. It is, therefore, possible that the PNP would dissolve in these ILs, but within the accessible time range of the simulations the rearrangements that are involved in the disintegration of the particle cannot be completed. For most ILs the *N* and *A* values fluctuate roughly around a constant value through the simulation (see Supp. Info.), suggesting that they have indeed reached an equilibrium state, and the data presented in Table 3 are physically realistic. The only exception was [C_8_C_1_Im][NTf_2_], in which case both quantities drifted slowly toward higher values, indicating an ongoing disintegration process.

**Figure 6:**
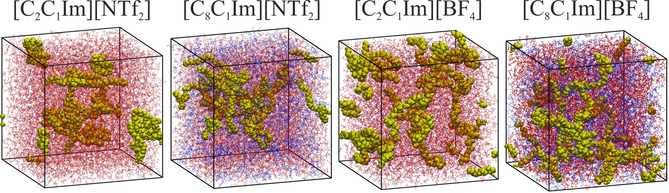
Simulation boxes containing the polyethylene chains (yellow) in the different ionic liquids (polar domain: red; non‐polar domain: blue), after 30 ns of simulation.

To overcome this problem, we performed further simulations with the [C_2_C_1_Im][NTf_2_], [C_8_C_1_Im][NTf_2_], [C_2_C_1_Im][BF_4_], and [C_8_C_1_Im][BF_4_] ILs, having the dissociated PE chains distributed randomly in the solution. In case of accurate sampling, the two sets of calculations – with the globular and disintegrated PNP – should converge into similar structures. The last snapshots of these simulations are presented in Figure 6, while the domain analysis data are shown in Table 4. The difference to the structures and data from the simulations with the PNP is striking. When plotting *N* and *A* against time, it becomes clear that the chains start to aggregate throughout the simulation in case of all ILs (see Supp. Inf.). These two sets of structures are possible to compare through their average potential energy over the last ns of the simulations, revealing which of the two states are closer to the preferential conformation of the system. According to the energies, the PNP is more stable in all ILs, except for [C_8_C_1_Im][NTf_2_]. Thus, according to the energetic data and the trends in the variations in *A* and *N* values, the PNP is supposed to avoid disintegration in all [BF_4_] ILs and in [NTf_2_] ILs with a shorter side chain. In contrast, in [C_8_C_1_Im][NTf_2_] the dissociated state is more stable, and therefore in this ionic liquid apparently the dissociation to individual chains should occur.

**Table 4:** Domain analysis for the systems containing the polyethylene chains. The number of polyethylene (*N* ^PE^), polar (*N* ^P^) and non‐polar domains (*N* ^N^), as well as the total outer surface area of polyethylene (viz. polyethylene‐solvent contact area, *A* ^PE^) are presented.

System	*N* ^PE^	*N* ^P^	*N* ^N^	*A* ^PE^ Å^2^	*A* ^PE*/*N^ Å^2^
[C_2_C_1_Im][NTf_2_]	3.5	1.0	681.2	21741.2	2525.0
[C_8_C_1_Im][NTf_2_]	3.7	1.0	3.7	25603.8	12978.1
[C_2_C_1_Im][BF_4_]	2.0	1.0	328.1	22402.0	4321.1
[C_8_C_1_Im][BF_4_]	4.0	1.0	1.3	22996.9	13633.6

**Figure 7:**
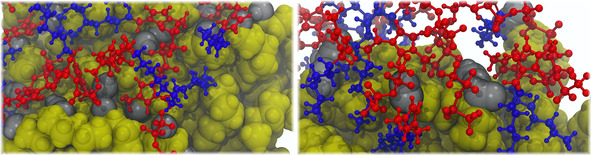
Aggregation of polar moieties of the IL at the hydroxyl (left) and formyl (right) substituents of partially oxidized polyethylene nanoparticles after 30 ns of MD simulation (yellow: polyethylene; silver: polar substituents on polyethylene; red: polar domain of the IL; blue: non‐polar domain of the IL).

**Figure 8:**
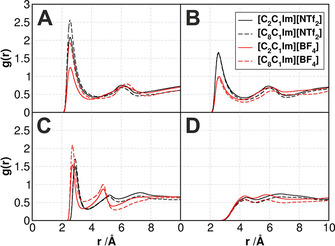
Radial distribution functions, representing the interactions of the ionic liquid solvents with the oxidized nanoplastic particle. **A**: interactions between the ‐OH oxygen atoms of the hydroxyl‐substituted polyethylene and the H2 hydrogen atom of the imidazolium ring; **B**: interactions between the −CHO oxygen atoms of the formyl‐substituted polyethylene and the H2 hydrogen atom of the imidazolium ring; **C**: interactions between the −OH oxygen atoms of the hydroxyl substituted polyethylene and the oxygen or fluorine atoms of the anions; **D**: interactions between the −CHO oxygen atoms of the formyl‐substituted polyethylene and the oxygen or fluorine atoms of the anions.

As mentioned above, we suggest that the amphiphilic nature and the corresponding microstructure in ILs is beneficial for the extraction of partially oxidized PNPs as well, since the ions that constitute the IL extractant can orient themselves at the surface of the PNP in a manner that they maximize the like‐like interactions. To verify this hypothesis, we modified the surface of the PNPs, and decorated it with polar groups, which are consistent with possible oxidation processes.^87^, ^88^ In one set of simulations ‐OH groups were added at 49 randomly chosen interfacial sites, while in another set ‐CHO substituents were introduced at the same sites. In case of the shorter side chains it is clearly possible for these polar groups to be covered by the polar domain, since the overwhelming majority of the liquid consists of the ionic moieties (see $r^{N}$ in Table 3). In case of the longer chains, however, one could argue that through the solvation of the PNP surface by the long alkyl groups of the IL cation the ionic moieties do not have access to the polar hydroxyl or formyl groups, and thereby their stabilization is not possible. According to our simulations, however, all these oxidized regions are covered with a high concentration of the polar domain of the IL (Figure 7). According to the radial distribution functions (RDFs, Figure 8A–B), the strongest^93^ hydrogen bond donor site of the imidazolium cation, at position 2 of the ring, can form hydrogen bonds with the oxygen atom of both functional groups considered here. Since the [BF_4_] anion is a significantly stronger hydrogen bond acceptor than the [NTf_2_], the ring hydrogen atoms of the latter kind of ILs are more accessible for interaction with the oxidized groups, shown by the higher *g*(*r*) values for the corresponding peaks. In fact, in case of the ‐CHO groups, the first peaks barely reach the $g\left(r\right)=1$ value, although it has to be mentioned here that due to the limited spatial accessibility of the sites – hindered by the plastic chains and the bulk of the PNP itself – these values can already by considered statistically relevant. Furthermore, the hydrogen bond donor capacity of the ‐OH groups attracts the anions (Figure 8C). In agreement with the trends in hydrogen bond acceptor strength, the [BF_4_] anions aggregate more at these sites. The RDF peaks indicate that the association of the polar moieties of the IL to the oxidized functional groups of the PNP is roughly independent of the side chain length, as the slight increase for the longer side chains can be partly attributed to the lower concentration of polar group in a unit volume of the IL, which causes through dilution effects an increase in the peak heights when calculating the *g*(*r*). Therefore, taking into consideration the high degree of oxidation in waste plastic,^87^, ^88^ ILs should be excellent solvents for PNP extraction from realistic environmental samples.

Regarding the phase transfer process, the side chains and the corresponding microheterogeneity raises further questions, which have to be considered. ILs are known to form distinct structures at interfaces, including those with water.^54^, ^94^, ^95^, ^96^, ^97^ At the aqueous interface, ILs present their polar moieties toward the water. Such a highly charged and polar interface could be considered as a barrier for the phase transfer of the non‐polar PNP, since it has to overcome the non‐favorable interactions with the polar groups before arriving in the stabilizing bulk IL media. For this reason, and to further understand the extraction process, we modeled the phase transfer of the PNP from an aqueous phase to an IL phase for two example ILs: [C_2_C_1_Im][NTf_2_] and [C_8_C_1_Im][NTf_2_].

**Figure 9:**
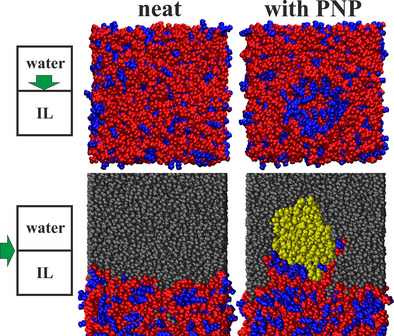
Domain structure of the [C_8_C_1_Im][NTf_2_]/water interface (blue: non‐polar; red: polar; yellow: polyethylene; grey: water; for the color definitions, see Figure 2). Structural rearrangement of the IL ions upon the presence of a polyethylene nanoparticle. Note that in the top right panel the plastic nanoparticle (omitted for visibility) changes the surface structure, making the predominantly polar surface mostly non‐polar through the rearrangement of IL ions.

Moving the particle slowly from one phase to the other, the structural changes that lead to the phase transfer can be tracked. In the beginning of the simulation, with the particle being far away from the interface within the bulk of the aqueous phase, the structure of the IL‐water interface can be observed. In [C_8_C_1_Im][NTf_2_], this region is structured in two ways: Firstly, the IL ions point their polar part toward the aqueous phase, to maximize the Coulombic interactions between the IL and water (Figure 9 upper left). Secondly, the microheterogeneity of the IL can be recognized already at this region, exhibiting also some non‐polar moieties directly underneath the first layer of the polar domain at the interface (Figure 9 lower left).

**Figure 10:**
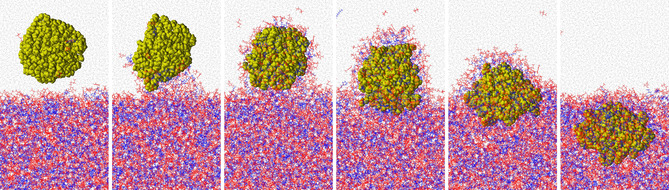
Phase transfer of a polyethylene nanoparticle from aqueous solution to [C_8_C_1_Im][NTf_2_] (blue: non‐polar; red: polar; yellow: polyethylene; grey: water; for the definitions, see Figure 2).

Already at a larger distance from the ionic liquid phase, the PNP attracts some [C_8_C_1_Im] cations, which are then adsorbed at its surface within the aqueous phase, forming a partial micelle, as expected from an amphiphilic molecule. As the particle is moved closer to the IL, the water‐IL interface is deformed, so that the water‐PE surface with an unfavorable mismatch of polar and non‐polar interactions can be minimized (see second panel from the left, Figure 10). As the PNP approaches the IL even more, the IL interface at the particle rearranges, so that the non‐polar moieties of the IL cations directly adjacent to the water/IL interface can get in contact with the polyethylene (Figure 9 upper and lower right). In other words, the interfacial IL cations reorient in a manner that they can point their non‐polar side chains toward the approaching plastic. In the light of the above results, if the polyethylene surface should contain any oxidized groups on its surface due to natural oxidation processes, at this point in the extraction process the PNP surface could act as a perfect template for the IL, and could trigger changes in the surface structure that are ideal for the stabilization and phase transfer of the plastic. Thus, the polar functional groups of the oxidized PNP could be solvated by the polar domain, while the non‐polar groups could exploit the presence of the non‐polar domain to facilitate the phase transfer. As the particle moves even closer to the IL (Figure 10), the ions start to surround it, forming a micelle, even before the PNP would be fully submerged within the IL phase. This encompassed structure moves deeper into the IL, and while the surface starts to reorder to resemble the original structure, the polymer chains of the particle begin their (partial) disentanglement, as described above.

Due to the lack of microheterogeneity in [C_2_C_1_Im][NTf_2_], the interface does not exhibit the aforementioned structural features, and thereby the transfer of the PNP does not involve such severe reorientation of ions. The shorter side chain of the cation results in the partial mixing of the two phases, i. e., a higher water content in the ionic liquid phase. This finding is in good agreement with earlier experiments on the phase separation behavior of IL/water mixtures.^44^

The energetics of the process can also be estimated through the present simulations. The free energy profile does not show any barriers with either of the two ILs considered here (see Supp. Inf.), which means that the phase transfer should not be kinetically hindered. This important result is in agreement with the structural changes observed above regarding the spontaneous reorientation of the IL ions at the interface upon the arrival of the PNP. In accordance with the findings above, which showed a more intensive IL‐PNP interplay in case of longer side chains at the imidazolium cation, the phase transfer is significantly more exothermic for [C_8_C_1_Im][NTf_2_] as extractant (−73 kcal mol^−1^) than for [C_2_C_1_Im][NTf_2_] (−12 kcal mol^−1^).

## Conclusion

In this study we suggest considering ionic liquids (ILs) as extractants for the removal of nanoplastics (PNPs) from aqueous solutions. By altering the length of the alkyl side chain of the IL, or by changing the anions, the strength of the interactions between the solvent and the PNP can be tuned. The polymer chains of the plastic particle partially disentagle into a loosely associated structure, in order to increase the contact surface area with the solvent. However, as opposed to non‐polar molecular solvents, the particle does not disintegrate into individual chains in most of the tetrafluoroborate and bis(trifluoromethylsulfonyl)imide ILs. The only exception was [C_8_C_1_Im][NTf_2_], showing that by varying the side chain length and the anion, the stability of the particles, i. e. their disintegration can be controlled and adjusted to the given aims. We also demonstrated that due to the amphiphilic nature of ILs, the interfacial polar groups of partially oxidized nanoplastics can be also solvated by the suitable building blocks of the IL: The non‐polar regions of the plastic can interact with the alkyl side chains, whereas the polar regions with the ionic head groups through hydrogen bonding. Accordingly, ILs should extract plastic nanoparticles (nanoplastics) with varying complex structures, which result from the interplay of these compounds with air, sunlight, and other environmental effects.

The interfacial microheterogeneous structure of [C_8_C_1_Im][NTf_2_] does not hinder the phase transfer. The layering of the interfacial region of the IL, presenting the polar regions of the extractant to the aqueous solution, is altered by the PNP. The cations at the interface flip, turning their alkyl chains toward the plastic in order to minimize unlike and maximize like interactions, resulting in a thermodynamically and kinetically favorable phase transfer. This reorientation also shows that if the surface plastic would be decorated with polar substituents or substances – through adsorption or oxidation processes – the IL surface could adjust to their presence as to a template, that both polar and non‐polar functionalities could benefit from the interaction with the extractant ions. The IL ions were observed to create a micelle around the PNP at the water/IL interface, stabilizing the plastic even before the phase transition is completed. These results clearly indicate that employing ILs can indeed lead to promising processes to handle nanoplastic contamination where it is most necessary.

## Conflict of interest

The authors declare no conflict of interest.

## Supporting information

As a service to our authors and readers, this journal provides supporting information supplied by the authors. Such materials are peer reviewed and may be re‐organized for online delivery, but are not copy‐edited or typeset. Technical support issues arising from supporting information (other than missing files) should be addressed to the authors.

Supplementary
