# Supplementary material for: Ionic Liquids as Extractants for Nanoplastics
Source: ChemSusChem. 2020 Sep 8;13(20):5449–59. doi: 10.1002/cssc.202001749 (PMC7692928; doi:10.1002/cssc.202001749)
Supplement: Supplementary file 1 — Supplementary [file CSSC-13-5449-s001.pdf]

ChemSusChem

Supporting Information

Ionic Liquids as Extractants for Nanoplastics

Roman Elfgen, Sascha Gehrke, and Oldamur Hollóczki*© 2020 The Authors. Published by Wiley-VCH GmbH. This is an open access article under the terms of the Creative Commons Attribution License, which permits use, distribution and reproduction in any medium, provided the original work is properly cited.

Supporting Information

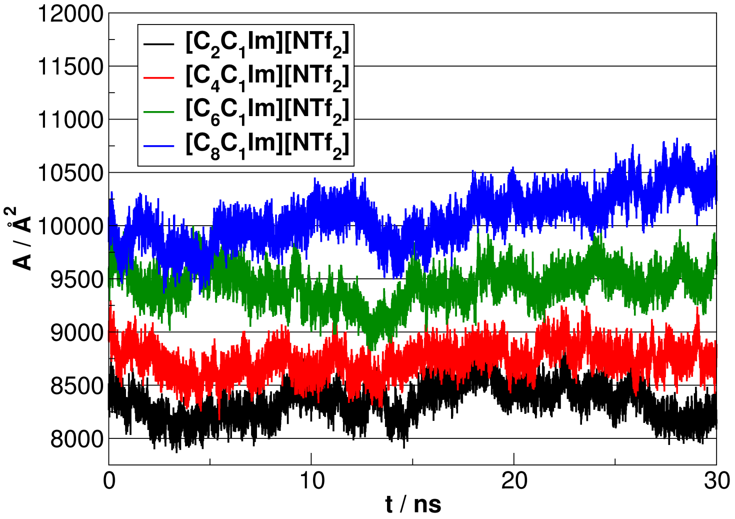

Figure 1: Changes in the surface area A of the PNP versus simulation time in NTf_2 ionic liquids, starting from the globular polyethylene PNP.

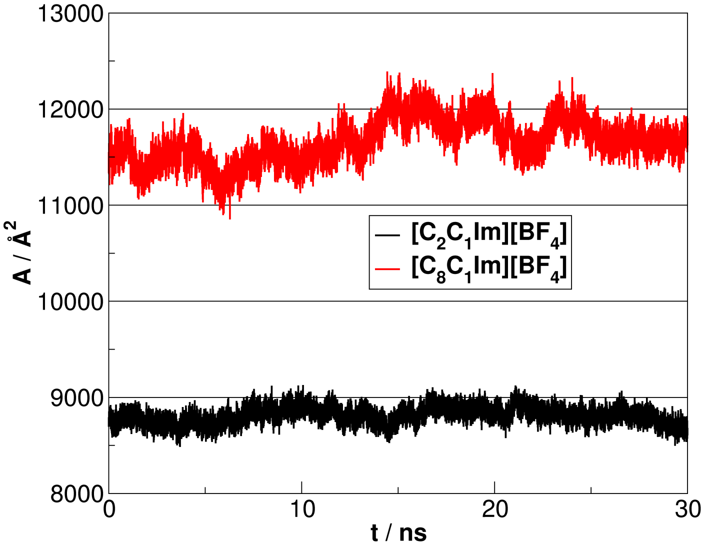

Figure 2: Changes in the surface area A of the PNP versus simulation time in BF_4 ionic liquids, starting from the globular polyethylene PNP.

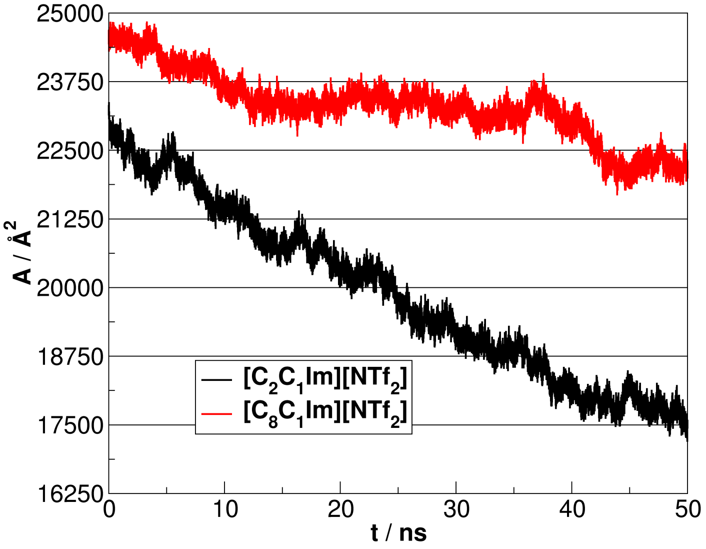

Figure 3: Changes in the surface area A of the PNP versus simulation time in NTf_2 ionic liquids, starting from the randomly distributed, dissociated chains.

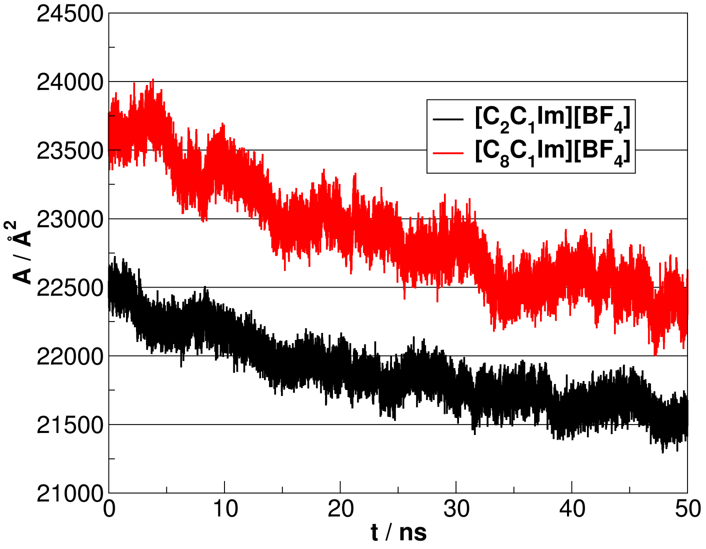

Figure 4: Changes in the surface area A of the PNP versus simulation time in BF_4 ionic liquids, starting from the randomly distributed, dissociated chains.

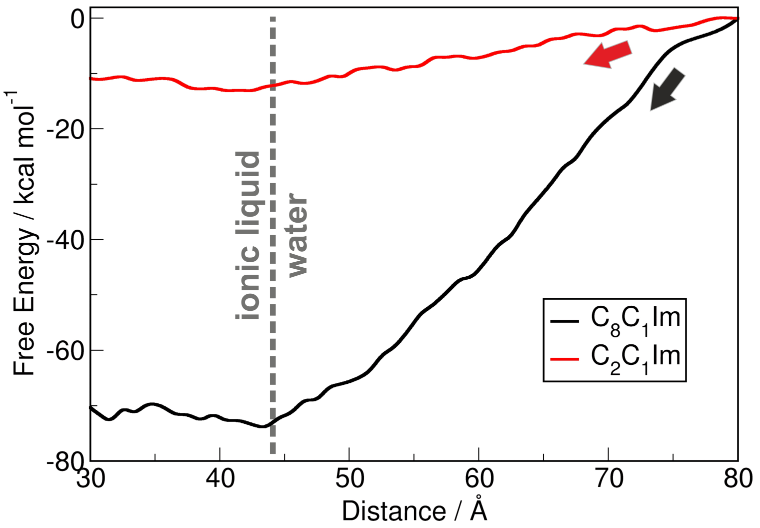

Figure 5: Free energy curves of the phase transfer of the PNP particle from the aqueous phase (right) to the ionic liquid phase (left), obtained from umbrella sampling simulations. The particle was initially situated in the aqueous phase, and gradually moved toward the center of mass of the ionic liquid phase, across the liquid-liquid interface. The location of the particle is defined by the Z component of the distance vector connecting the center of mass of the plastic and that of the ionic liquid phase. The particle crosses the interface at a distance of ca. 44 Å.
